# Efficacy of statins in patients with diabetic nephropathy: a meta-analysis of randomized controlled trials

**DOI:** 10.1186/s12944-016-0350-0

**Published:** 2016-10-12

**Authors:** Xue Shen, Zhongwen Zhang, Xiaoqian Zhang, Junyu Zhao, Xiaojun Zhou, Qinglei Xu, Hongxia Shang, Jianjun Dong, Lin Liao

**Affiliations:** 1 Department of Medicine, Shandong Provincial Qianfoshan Hospital, Shandong University of Traditional Chinese Medicine, Jinan, 250014 China; 2 Department of Medicine, Division of Endocrinology, Shandong Provincial Qianfoshan Hospital, Shandong University, No.16766, Jingshi Road, Lixia District, Jinan, 250014 Shandong Province China; 3 Department of Medicine, Division of Endocrinology, Qilu Hospital of Shandong University, Jinan, 250012 China

**Keywords:** Statins, Diabetic Nephropathy, Meta-analysis

## Abstract

**Background:**

The effects of statins in patients with diabetic nephropathy are controversial. With increasing interest in the potential therapeutic role of statins in diabetic nephropathy, it is essential to evaluate its real effects.

**Methods:**

PubMed, EMBASE, Web of Science databases, Cochrane Central Register of Controlled Trials and China National Knowledge Infrastructure were systematically searched for randomized controlled trials (RCTs) of statins in patients with diabetic nephropathy.

**Results:**

Fourteen trials with 2866 participants were included in our meta-analysis. Compared with placebo, albuminuria and urinary albumin excretion rates in the statin group were reduced by 0.46 [95 % confidence interval (CI),−0.68 to −0.25, *P* < 0.0001] and 1.68 (95 % CI, −3.23 to −0.12, *P* = 0.03), respectively. The reduction of albuminuria was greater in patients of type 2 diabetes mellitus with diabetic nephropathy [standardized mean difference (SMD), −0.56; 95 % CI, −0.80 to −0.32, *P* < 0.00001] and the decrease was significant during the 1 to 3 years period of statin therapy (SMD, −0.57; 95 % CI, −0.95 to −0.19, *P* = 0.003). Subgroup analysis demonstrated the effects of statins were much stronger in subjects with pathologic albuminuria: change of −0.71 (95 % CI, −1.09 to −0.33, *P* = 0.0003) for those with urinary protein excretion 30 to 300 mg/day, −0.37 (95 % CI, −0.67 to −0.06, *P* = 0.02) for those with excretion more than 300 mg/day and −0.29 (95 % CI, −0.78 to 0.21, *P* = 0.26) for those with excretion less than 30 mg/day. In contrast, statins did not significantly reduce estimated glomerular filtration rate, serum creatinine and blood urea nitrogen levels.

**Conclusions:**

Statins decrease the albuminuria and urinary albumin excretion rates significantly. The efficacy of statins on renal function is time dependent and better in type 2 diabetic patients with nephropathy.

## Background

According to the International Diabetes Federation [1], it is projected that the number of people with diabetes worldwide will increase from 382 million in 2013 to 592 million by 2035. Diabetic nephropathy (DN) is one of the most common and serious chronic complication of diabetes and it is the leading cause of end-stage renal disease [2]. However, beyond angiotensin II-receptor blockers (ARB) and angiotensin-converting enzyme inhibitors (ACEI), therapeutic options to block the progression of diabetic nephropathy are limited and other strategies to preserve kidney function are needed.

A number of potential mechanisms for kidney damage in DN have been identified. Hyperlipidemia may play an important role in the progression of DN and it may impair the messangial cells through its lipotoxicity or by promoting intrarenal atherosclerosis [3–5]. Statin, 3-hydroxy-3 methylglutaryl coenzyme A (HMG CoA) reductase inhibitor, is a kind of antihyperlipidemic drug that used worldwide for its strong low-density lipoprotein cholesterol (LDL-C)-lowering effects and established safety. Recently, there are growing studies suggested that statins may offer renoprotective effects and beneficial effect on pathologic albuminuria and decrease the reduction of estimated glomerular filtration rate (eGFR) [6–8]. However, some trials [9, 10] failed to demonstrate that statin improve eGFR.

To assess whether statins have beneficial effects on renal outcomes in diabetic nephropathy, we performed this meta-analysis to investigate the potential therapy of statins in patients with diabetic nephropathy.

## Methods

### Literature search

We conducted a search of PubMed, EMBASE, Web of Science databases, Cochrane Central Register of Controlled Trials and China National Knowledge Infrastructure (CNKI). All relevant articles were published in English and Chinese. The following Medical Subject Headings (MeSH) and text words were used: Hydroxymethylglutaryl-CoA reductase inhibitors, atorvastatin, simvastatin, rosuvastatin, pravastatin, lovastatin, fluvastatin, cerivastatin, mevastatin, pitavastatin, statin, kidney, renal, diabetic nephropathy, randomized controlled trial (RCT), controlled clinical trial and random allocation. We also searched the additional trials at the trial register centres (http://www.clinicaltrials.gov). Clinical trials were included if the following criterias were met: (1) Primary study of statins versus control (placebo or usual care); (2) Diabetic nephropathy patients with type 1 and type 2 diabetes mellitus at least 18 years old without pregnancy; (3) Patients with diabetic nephropathy in experimental group were defined as those who used statins, regardless of dosages, mode of administration or treatment duration; (4) RCT design; (5) Report of baseline and the end of follow-up data on renal function [estimated glomerular filtration rate (eGFR), urinary albumin excretion rates (UAER), serum creatinine (Scr), blood urea nitrogen (BUN) or albuminuria). Exclusion criteria included: (1) Kidney damage due to diseases other than type 1 or type 2 diabetes. (2) The final stage of diabetic nephropathy or end-stage-renal disease (ESRD), defined as onset of renal replacement therapy or death attributed to diabetic nephropathy.

### Study selection and data extraction

Two reviewers independently screened abstracts according to the inclusion criteria, and disagreements between reviewers were resolved by consensus. We developed a data extraction sheet based on the Cochrane Consumers and Communication Review Group’s data extraction template. One reviewer extracted the following data from included studies and the second reviewer verified the extracted data. Disagreements were resolved by discussion between the two reviewers. If an agreement could not be reached between two reviewers, a third author would decide. Information extracted included: (1) characteristics of trial subjects (including age, sex ratio, duration of diabetes and baseline value of renal function) and the trial’s inclusion/exclusion criteria; (2) type of intervention (including dosage, duration and frequency); and (3) type of outcome and measurement.

### Statistical analysis

The primary outcome was the change of albuminuria from the baseline. Other outcomes include: change from baseline in eGFR, UAER, Scr, BUN. The meta-analysis with fixed-model or random-model was performed by weighted mean difference (WMD), standardized mean difference (SMD) and 95 % confidence interval (CI) for outcome of continuous variables. Subgroup analysis by characteristics of patients (i.e., ethnicity, stage of diabetes nephropathy) and study design (i.e., whether ACEI/ARB was used or not) were performed. *I* ^*2*^ was calculated as an index of heterogeneity between studies. If *I* ^*2*^ was higher than 50 %, the sensitive analysis should be performed to find out the source of heterogeneity and to assess whether the results could be significantly influenced.

### Quality assessment and publication bias

Study quality and bias risk were assessed via predefined categories: randomization, allocation concealment, quality of blinding (participants,personnel and outcome assessment), withdrawal and loss and reporting bias. Two reviewers independently determined these items. Sensitive analysis was performed in studies with low quality. The analyses were performed using Review Manager 5.2 (Cochrane Collaboration, http://www.cochrane.org).

## Results

### Search results and study characteristics

**Fig. 1:**
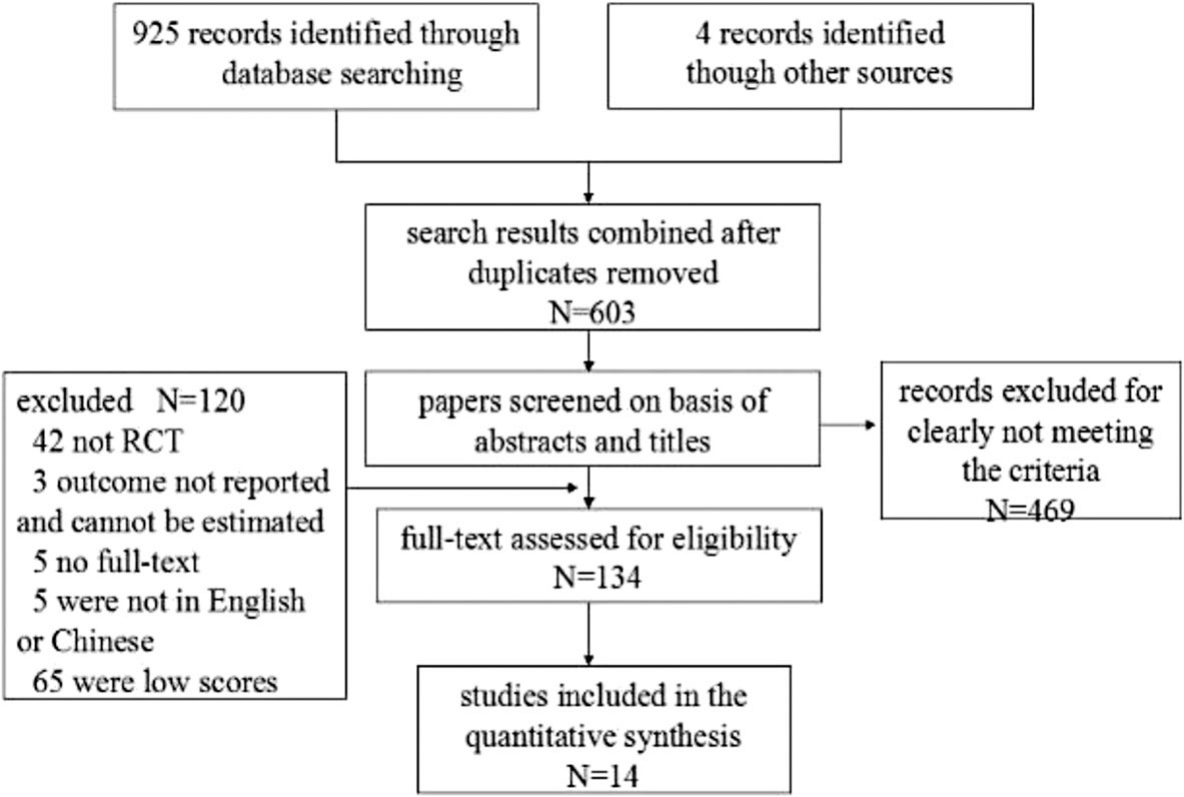
Flow diagram of study selection

**Fig. 2:**
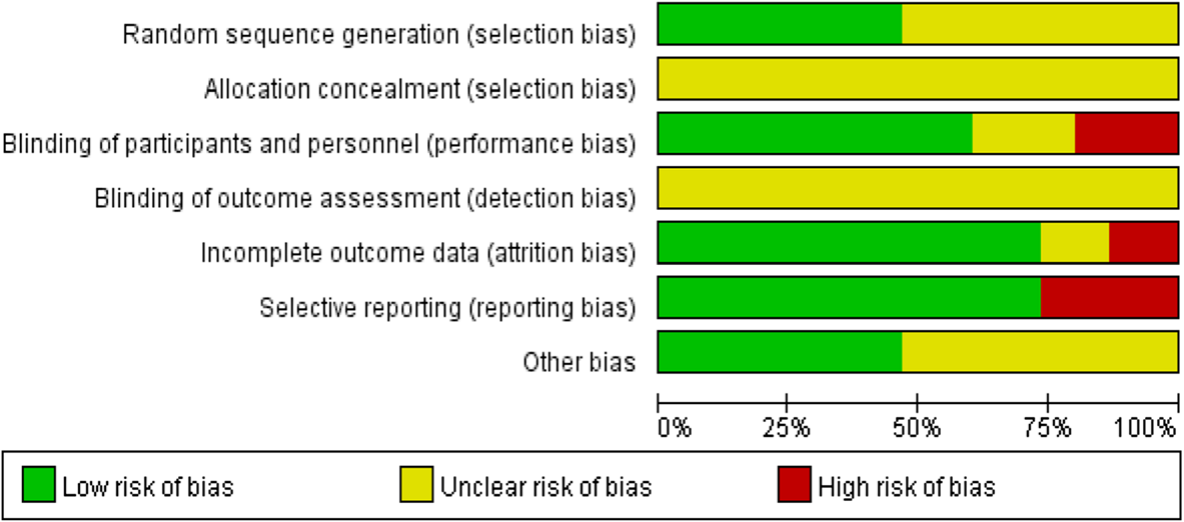
Methodological quality of the included studies

Initially, 929 potentially relevant articles were identified. After adjusting for duplicates, 603 studies remained, consisting of 134 potentially relevant studies and 469 studies that were excluded after reviewing titles and abstracts. Of 134 potentially relevant studies, 120 failed to match the inclusion criteria. Finally 14 articles [9–22] with a total of 2866 participants were included in this meta-analysis (Fig. 1). Of these, 3 studies [20–22] were reported in Chinese, 11 were in English [9–19]. Among the final 14 studies, 7 were conducted in Caucasians and others in Asians. The most commonly diabetic nephropathy included in this meta-analysis was diabetic nephropathy with type 2 diabetes mellitus. Ten studies provided data of albuminuria, 5 of eGFR, 5 of UAER, 4 of Scr and 2 of BUN. Eight different statins involved in this study, including simvastatin [12, 13, 15, 17, 22], atorvastatin [9, 11, 20, 21], pitavastatin [18], lovastatin [14], cerivastatin [19], rosuvastatin [10] and pravastatin [16]. The study period ranged from approximately 3 months to 2 years. ACEI or ARB were used in studies, except for 3 studies [11, 15, 17]. The characteristics of the included studies were shown in Tables 1 and 2. Among them, 7 studies [9, 10, 12, 14, 20–22] mentioned the specific randomized method, the others referred “random” but did not mention the detail. Nine studies [9, 11–13, 15–19] were double-blinded. Detail was shown in Fig. 2.

**Table 1:** Fifteen randomized, placebo-controlled trials assessing the effect of statins on renal outcomes in diabetic nephropathy

Studies	Country	Intervention	Sample sizes (n)		Use of ACEI or ARB (%)		Mean age (years)		Duration of diabetic nephropathy, (years)		Follow-up (months)
			statin	control	statin	control	statin	control	statin	control	
CARDS 2009 [9]	UK	Atorvastatin, 10 mg/d	1154	1159	44.6	43.6	61.5	61.8	**—**	**—**	24
Masanori 2011 [10]	Japan	Rosuvastatin, 2.5–10 mg/d	52	52	100	100	64.5	64.9	**—**	**—**	6
Dalla 2003 [11]	Italy	Atorvastatin 10 mg/d	12	13	0	0	66	63	10	9	12
Linda 2001 [12]	USA	Simvastatin, 10 mg/d	19	20	5	15	33.3	31.0	22.8	20.8	18
E. Hommel 1992 [13]	Denmark	Simvastatin, 10–20 mg/d	12	9	67	89	41	35	27	27	3
Lam 1995 [14]	China	Lovastatin, 30 mg/d	16	18	12.5	16.7	58.9	53.9	**—**	**—**	24
S.Nielsen 1993 [15]	Denmark	Simvastatin 10–20 mg/d	8	10	0	0	65	65	10.2	10.9	9
Zhang 1995 [16]	Belgium	Pravastatin, 20 mg	10	10	**—**	**—**	43	43	**—**	**—**	3
Giancarlo 1997 [17]	Italy	Simvastatin, 20 mg/d	10	9	0	0	60	62	**—**	**—**	12
Tsukasa 2005 [18]	Japan	Pitavastatin, 1 mg/d	10	10	**—**	**—**	51	49	13	12	12
Tsukasa 2001 [19]	Japan	Cerivastatin, 0.15 mg/d	30	30	**—**	**—**	58	55	**—**	**—**	6
Wu 2013 [20]	China	Atorvastatin, 20 mg/d	39	39	100	100	55.15	55.33	5.18	4.82	6
Du 2015 [21]	China	Atorvastatin, 20 mg/d	26	26	100	100	56	57	10	10	3
Xiang 2005 [22]	China	Simvastatin, 20 mg/d	32	31	100	100	50	49	15	14	6

*USA* the United States of America, *UK* United Kingdom, *T2DM* type 2 diabetes mellitus, *T1DM* type 1 diabetes mellitus, **—**:not report

**Table 2:** Characteristics of the 14 randomized controlled trials Included in the meta-analysis

Study or author	Baseline LDL-C Level, (mg/dl)	Change in LDL-C^a^(mg/dl)	Baseline HDL-C Level,(mg/dl)	Change in HDL-C^a^,(mg/dl)	Baseline Triglyceride Level, (mg/dl)	Change in Triglyceride^a^,(mg/dl)
CARDS 2009 [9]	**—**	**—**	**—**	**—**	**—**	**—**
Masanori 2011 [10]	137	−54	49	+4	162	−32
Dalla 2003 [11]	149	−41	55	+1	162	−32
Linda 2001 [12]	125.5	−28.3	50.9	+2.2	76	−9.5
E. Hommel 1992 [13]	162.54	−61.92	57.7	+1.55	120.5	+11.52
Lam 1995 [14]	166.4	−50.31	42.57	−0.39	194.92	−17.72
S.Nielsen 1993 [15]	170.28	−58.05	48.76	−0.39	204.7	−20.4
Zhang 1995 [16]	123	−23	62	+1	105	−12
Giancarlo 1997 [17]	181.89	−54.18	50.31	0	141.8	−26.6
Tsukasa 2005 [18]	**—**	**—**	**—**	**—**	**—**	**—**
Tsukasa 2001 [19]	208	−62	22	+16	202	−42
Wu 2013 [20]	171.05	−61.15	**—**	**—**	225.9	−65.6
Du 2015 [21]	**—**	**—**	**—**	**—**	**—**	**—**
Xiang 2005 [22]	166.41	−19.35	54.18	+7.74	221.5	−17.72

^a^In statin group;**—**:not report

### Quantitative data analysis

#### Effect of statins on albuminuria

**Fig. 3:**
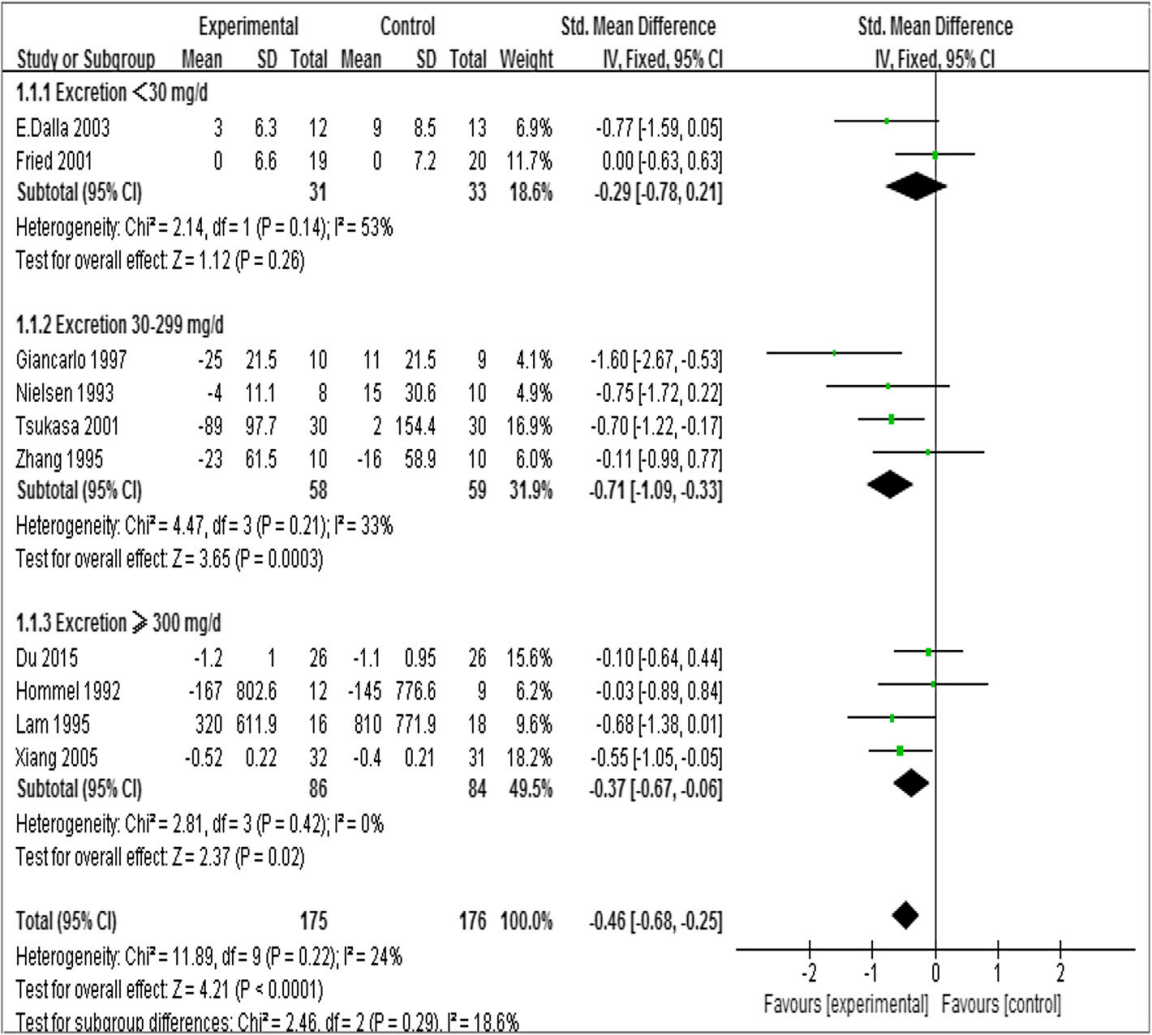
Forest plots of albuminuria after statins or placebo therapy in patients with albuminuria <30 mg/day, 30 to 300 mg/day, and >300 mg/day

Pooled data from 10 studies [11–17, 19, 21, 22] (20 groups) showed a statistical decrease in albuminuria compared with that in control group (SMD, −0.46; 95 % CI, −0.68 to −0.25; *P* < 0.0001), and the standardized mean difference in change from baseline was −0.71 (95 % CI, −1.09 to −0.33; *P* = 0.0003; *I* ^*2*^ = 33 %) for those with excretion of 30 to 299 mg/d; and −0.37 (95 % CI, −0.67 to −0.06; *P* = 0.02; *I* ^*2*^ = 0 %) for those with excretion of 300 or greater (Fig. 3). Although the statins were not the same subtype, there was no significant heterogeneity among all trials in our study (*I* ^*2*^ = 24 %).

**Fig. 4:**
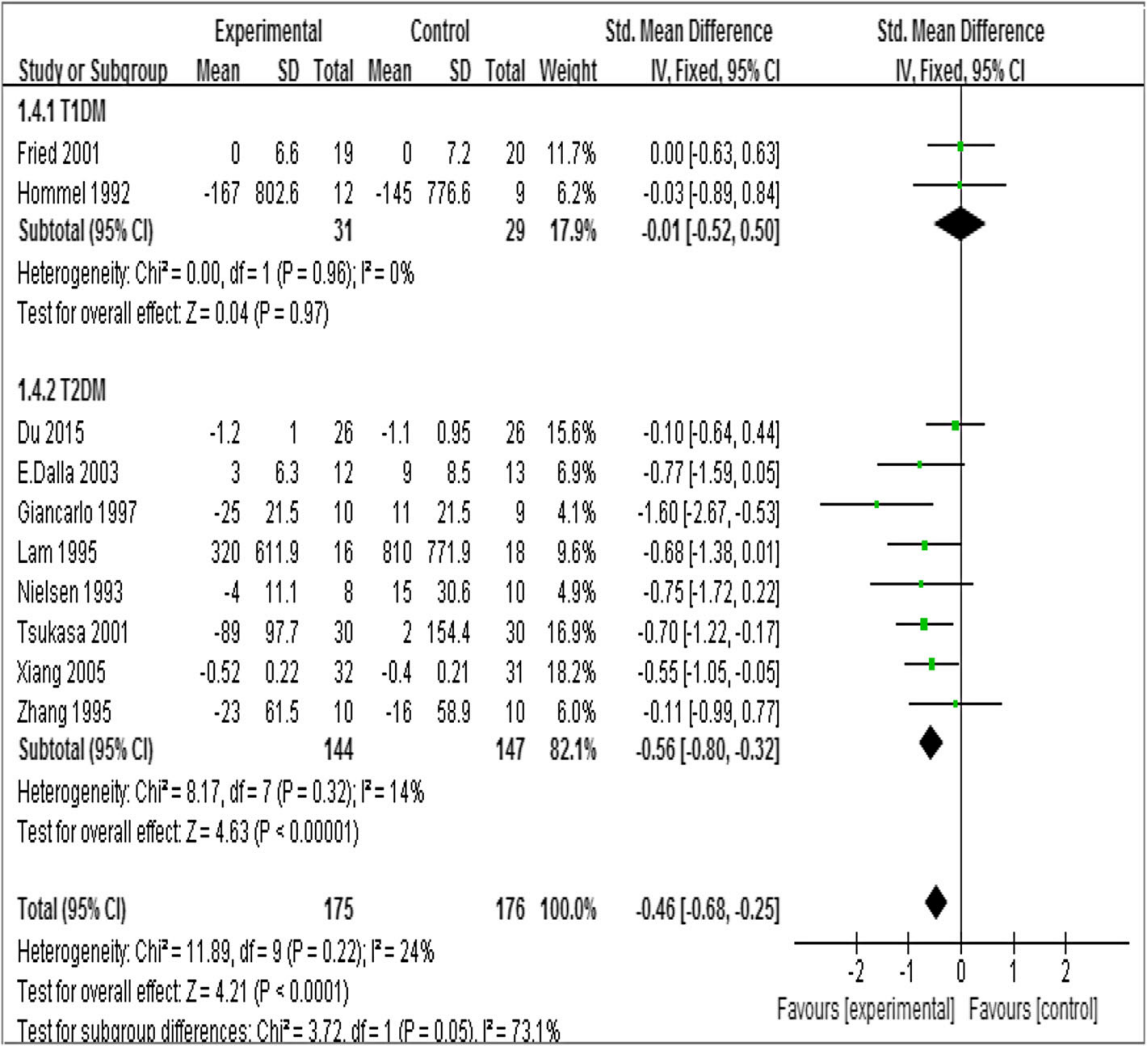
Forest plots of albuminuria after statins or placebo therapy in nephropathy patients with T1DM or T2DM

Statin did not decrease the urine albumin for those with excretion less than 30 mg/d (SMD,–0.29; 95 % CI,–0.78 to 0.21; *P* = 0.26, Fig. 3). In statin treated group, there was statistically significant reduction in albuminuria in the T2DM with diabetic nephropathy (SMD,–0.56; 95 % CI,–0.80 to −0.32; *P* < 0.00001, *I* ^*2*^ = 14 %), while no significant improvement in diabetic nephropathy of T1DM subgroup (SMD, −0.11; 95 % CI, −0.52 to 0.50; *I* ^*2*^ = 0 %; *P* = 0.97, Fig. 4). A greater decrease in albuminuria was observed in patients received statin therapy for 1 to 3 years (SMD, −0.57; 95 % CI, −0.95 to −0.19, *P* = 0.002) compared with those <1 year (SMD;–0.41, 95 % CI,–0.67 to −0.15, *P* = 0.003; Table 3). However, there was no significant difference between Asians (SMD −0.46, 95 % CI −0.80 to −0.12) and Caucasians population (SMD −0.54, 95 % CI −0.82 to −0.27; Table 3).

**Table 3:** Meta-analysis of the effect of statins on renal outcomes in diabetes

		Subjects	Heterogeneity			
Category	N	cases/controls	Ph	*I* ^*2*^(%)	SMD(95 % CI)	Z test
Albuminuria						
1. Overall	10	175/176	0.22	24	−0.46(−0.68,–0.25)	z = 4.21,p_z_ < 0.0001
2. Adjustment by ethnicity						
Caucasian	6	71/71	0.12	43	−0.41(−0.75,–0.07)	z = 2.36,p_z_ = 0.02
Asian	4	104/105	0.41	0	−0.50(−0.77,–0.22)	z = 3.51,p_z_ = 0.0004
3. Adjustment by subtypes of diabetes with diabetic nephropathy						
T1DM	2	31/29	0.96	0	−0.01(−0.52,0.50)	z = 0.04,p_z_ = 0.97
T2DM	8	144/147	0.31	14	−0.56(−0.80,–0.32)	z = 4.63,p_z_ < 0.00001
4. Adjustment by baseline of albuminuria						
< 30 mg/d	2	31/33	0.14	53	−0.29(−0.78,0.21)	z = 1.12,p_z_ = 0.26
30-299 mg/d	4	58/59	0.21	33	−0.71(−1.09,–0.33)	z = 3.65,p_z_ = 0.0003
≥ 300 mg/d	4	86/84	0.42	0	−0.37(−0.67,–0.06)	z = 2.37,p_z_ = 0.02
5. Adjustment by treatment duration						
< 1 year	6	118/116	0.50	0	−0.41(−0.67,–0.15)	z = 3.09,p_z_ = 0.002
1 ~ 3 years	4	57/60	0.07	58	−0.57(−0.95,–0.19)	z = 2.94,p_z_ = 0.003
6. Adjustment by dose of statins						
low-intensity statins	3	61/63	0.18	41	−0.48(−0.84,–0.12)	z = 2.61,p_z_ = 0.009
moderate-intensity statins	2	20/19	0.27	16	−0.35(−0.99,0.30)	z = 1.05,p_z_ = 0.29
high-intensity statins	5	94/94	0.13	44	−0.47(−0.77,–0.18)	z = 3.15,p_z_ = 0.002
eGFR						
1. Overall	6	1252/1257	0.73	0	0.49(−0.06,1.03)	z = 1.75,p_z_ = 0.08
2. Adjustment by ethnicity						
Caucasian	5	1184/1187	0.48	0	0.48(−0.08,1.04)	z = 1.69,p_z_ = 0.09
Asian	1	68/70	0.75	0	0.64(−1.87,3.15)	z = 0.50,p_z_ = 0.62
3. Adjustment by subtypes of diabetes with diabetic nephropathy						
T1DM	1	12/9	not applicable		−3.00(−17.22,11.22)	z = 0.41,p_z_ = 0.68
T2DM	5	1240/1248	0.64	0	0.49(−0.05,1.04)	z = 1.77,p_z_ = 0.08
4. Adjustment by baseline of albuminuria						
< 30 mg/d	1	913/918	not applicable		0.34(−0.28,0.96)	z = 1.08,p_z_ = 0.28
30-299 mg/d	4	311/312	0.52	0	1.03(−0.16,2.21)	z = 1.70,p_z_ = 0.09
≥ 300 mg/d	1	28/27	0.56	0	0.73(−6.15,7.61)	z = 0.21,p_z_ = 0.83
5. Whether combined with ACER or ARB						
with ACEI or ARB	4	1218/1220	0.74	0	0.04(−0.04,0.12)	z = 0.89,p_z_ = 0.37
without ACEI or ARB	2	18/20	0.94	0	1.14(0.44,1.84)	z = 3.20,p_z_ = 0.001
6. Adjustment by treatment duration						
< 1 year	3	72/71	0.47	0	0.69(−1.85,3.24)	z = 0.53,p_z_ = 0.59
1 ~ 3 years	3	1180/1186	0.56	0	0.48(−0.08,1.04)	z = 1.68,p_z_ = 0.09
7. Adjustment by dose of statins						
low-intensity statins	3	1206/1211	0.81	0	0.42(−0.13,0.98)	z = 1.51,p_z_ = 0.13
moderate-intensity statins	2	20/19	0.27	19	3.07(−6.28,12.43)	z = 0.64,p_z_ = 0.52
high-intensity statins	1	26/27	0.80	0	2.76(−0.83,6.36)	z = 1.51,p_z_ = 0.13
8. Adjustment by baseline of eGFR						
60 ~ 89 ml/min/1.73 m^2^	4	1234/1238	0.94	0	0.43(−0.12,0.98)	z = 1.52,p_z_ = 0.13
> =90 ml/min/1.73 m^2^	2	18/19	0.48	0	3.45(−0.40,7.30)	z = 1.76,p_z_ = 0.08
UAER						
1. Overall	5	99/100	<0.00001	93	−1.68(−3.23,–0.12)	z = 2.12,p_z_ = 0.03
2. Adjustment by ethnicity						
Caucasian	2	18/20	<0.00001	96	−25.99(−78.54,26.57)	z = 0.97,p_z_ = 0.33
Asian	3	81/80	0.0001	89	−1.78(−2.98,–0.57)	z = 2.88,p_z_ = 0.004
3. Adjustment by baseline of albuminuria						
30-299 mg/d	4	67/69	<0.00001	94	−2.17(−4.56,0.23)	z = 1.77,p_z_ = 0.08
≥ 300 mg/d	1	32/31	not applicable		−1.12(−1.65, −0.58)	z = 4.11,p_z_ < 0.0001
4. Adjustment by treatment duration						
< 1 year	4	89/90	<0.00001	95	−1.90(−3.91,0.11)	z = 1.85,p_z_ = 0.06
1 ~ 3 years	1	10/10	not applicable		−1.29(−2.28,–0.31)	z = 2.58,p_z_ = 0.010
5. Adjustment by dose of statins						
low-intensity statins	1	10/10	not applicable		−1.29(−2.28,–0.31)	z = 2.58,p_z_ = 0.010
moderate-intensity statins	1	8/10	not applicable		−53.77(−73.67,–33.88)	z = 5.30,p_z_ < 0.00001
high-intensity statins	3	81/80	<0.00001	93	−1.39(−2.87,0.09)	z = 1.84,p_z_ = 0.07
*Scr*						
1. Overall	4	127/126	<0.00001	95	0.75(−0.52,2.03)	z = 1.15,p_z_ = 0.25
2. Adjustment by baseline of albuminuria						
30-299 mg/d	2	69/69	<0.00001	98	1.62(−1.91,5.15)	z = 0.90,p_z_ = 0.37
≥ 300 mg/d	2	58/57	0.93	0	−0.05(−0.42,0.31)	z = 0.28,p_z_ = 0.78
4. Adjustment by dose of statins						
low-intensity statins	1	30/30	not applicable		3.44(2.62,4.25)	z = 0.00,p_z_ = 1.00
high-intensity statins	3	97/96	0.93	0	−0.10(−0.38,0.19)	z = 0.98,p_z_ = 0.33
BUN						
1.Overall	2	51/52	0.88	0	−0.26(−0.64,0.13)	z = 1.29,p_z_ = 0.20

*Abbreviations*: *N* number of involved studies, *Ph* P values for heterogeneity of Q test, *p*_*z*_ *<0.05* indicate significant association, *eGFR* estimated Glomerular Filtration Rate, *T1DM* type 1 diabetes mellitus, *T2DM* type 2 diabetes mellitus, *ACEI* angiotensin-converting enzyme inhibitors, *ARB* Angiotensin II -receptor blockers, *UAER* urinary albumin excretion rates, *Scr* serum creatinine, *BUN* blood urea nitrogen

Therefore, the results suggested that statins can reduce albuminuria significantly in patients of T2DM with diabetic nephropathy. And the beneficial effect of statins on renal function are significantly better in those with statin therapy longer than one year than that of less than one year.

#### Effect of statins on estimated glomerular filtration rate (eGFR)

Six studies [9, 10, 13–15, 17] enrolled, including 2509 participants with eGFR >60 ml/min/1.73 m^2^. Statins did not improve eGFR significantly in most studies and the change in the SMD of eGFR was 0.49 (95 % CI, −0.06 to 1.03, *P* = 0.08, *I* ^*2*^ = 0 %). Furthermore, different types of diabetic nephropathy, ethnicity, baseline of eGFR, treatment duration and dose of statins also did not influence eGFR in patients with statin and control therapy (Table 3).

#### Effect of statins on urinary albumin excretion rates (UAER)

**Fig. 5:**
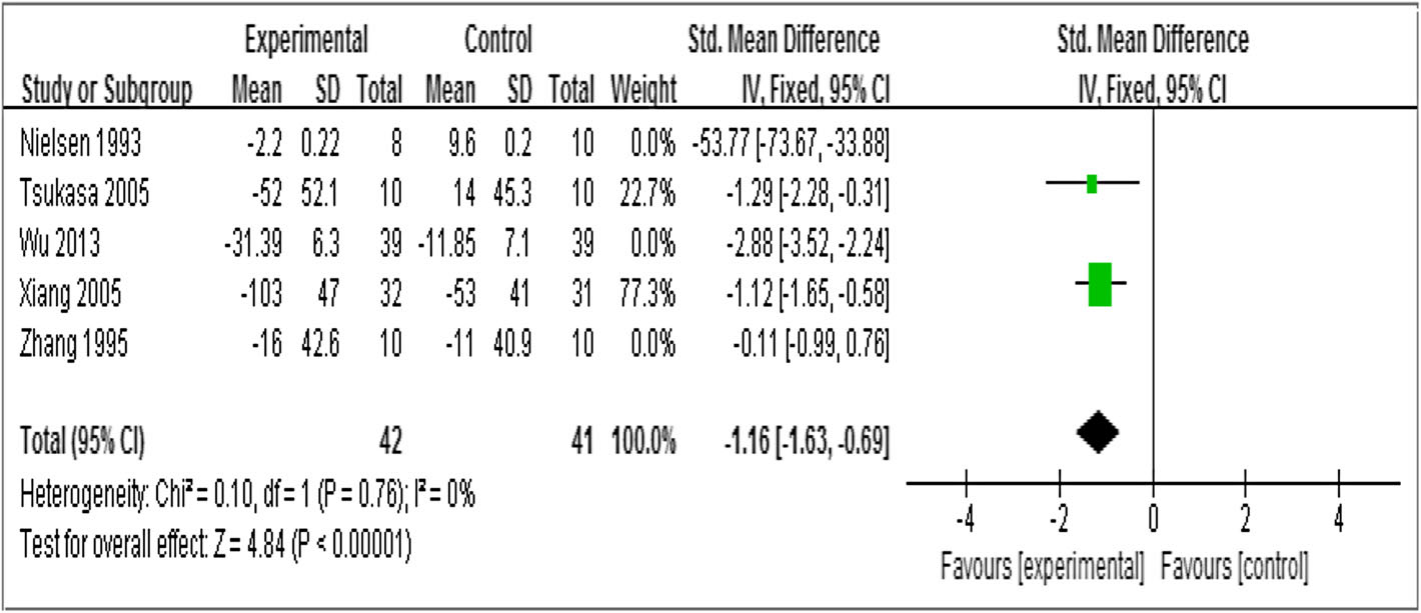
Forest plots of UAER (ug/min) for statins versus placebo in patients with diabetic nephropathy

The effect of statins on UAER was favorable in 5 studies [15, 16, 18, 20, 22]. Overall, the change in the SMD for UAER was −1.68 (95 % CI, −3.23 to −0.12, *P* = 0.03; Fig. 5) which indicated that compared with placebo, there was statistically significant reduction in UAER in statin-treated group. Because of the heterogeneity (heterozygosity test, Chi^2^ = 56.01, *P* < 0.00001, *I* ^*2*^ = 93 %), we conducted a subgroup analysis and sensitivity analysis. In subgroup analysis of ethnicity, stage of diabetic nephropathy, treatment duration and dose of statins, the heterogeneity still existed (Table 3). When the trials by Nielsen et al. (1993) [15], Zhang et al. (1995) [16] and Wu et al. (2013) [20] were removed, the heterogeneity disappeared (the *I* ^*2*^ reduced from 93 % to 0 %, *P* from <0.00001 to 0.76). The fixed-effect model was used to merge SMD values and the pooled SMD was −1.16 (95 % CI,–1.63 to −0.69, *P* < 0.00001), which indicated that statins reduced UAER significantly in patients with DN.

#### Effect of statins on serum creatinine (Scr)

We identified 4 randomized controlled trials [19–22] (253 participants) for the effect of statins on Scr. Because of the heterogeneity (heterozygosity test, Chi^2^ = 64.91, *P* <0.00001, *I* ^*2*^ = 95 %), we removed 1 study at a time to identify the source of heterogeneity. When the Study by Tsukasa et al. (2001) [19] was removed, the heterogeneity was no longer existence, which showed that the heterogeneity may came from the country difference of patients in the study. The fixed-effect model was used to merge SMD values and the pooled SMD was −0.10 (95%CI,–0.38 to 0.19; *P* = 0.50; Table 3), which means statins may have no effect on the Scr in patients with DN.

#### Effect of statins on blood urea nitrogen (BUN)

The result showed that there was no statistically difference in BUN in statin group compared with that in control group (SMD, −0.26; 95 % CI, −0.64 to 0.13; *P* = 0.20) and the heterogeneity among trials was not significant (*I* ^*2*^ = 0 %, Table 3).

### Assessment of publication bias

**Fig. 6:**
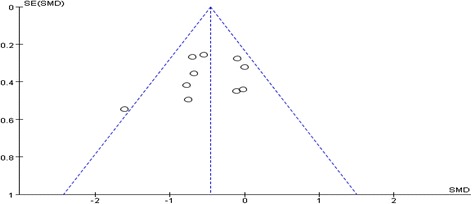
Funnel plot of publication bias for the effects of statins on renal outcomes in diabetic nephropathy

Publication biases were examined by funnel plot and no significant publication bias was found among studies included in our meta-analysis (Fig. 6).

### Subgroup and sensitivity analysis

**Fig. 7:**
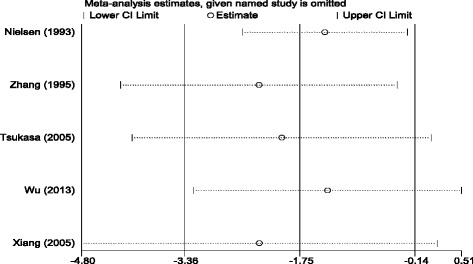
Sensitivity analysis for the efficacy of statins on UAER in patients with diabetic nephropathy

Because of study heterogeneity, subgroup and meta-regression analyses were conducted by ethnicity, baseline of albuminuria, treatment duration, and dose of statins (Table 3). However, the results of statin on renal function were not influenced. In consideration of the different baseline of UAER, sensitivity analysis was performed to assess the impact of every study on the overall conclusions. After the three trials by Nielsen et al. (1993) [15], Zhang et al. (1995) [16] and Wu et al. (2013) [20] were eliminated, the heterogeneity test (the *I* ^*2*^ reduces from 93 % to 0 %, *P* from <0.00001 to 0.76) indicated that baseline of UAER may be a source of heterogeneity (Fig. 7).

## Discussion

Statins are frequently used to improve lipid profile and they are also reported to reduce cardiovascular events [23], albuminuria [6] and diabetic glomerulosclerosis in experimental animals [24]. However, the efficacy of statins in improving renal function in patients with diabetic nephropathy is still controversial. To answer this question, we did this study and found that statins could reduce the albuminuria and UAER compared with that in control groups. The beneficial effect of statins on renal function is time dependent and better in type 2 diabeties with nephropathy.

As we all know, the degree of albuminuria is a risk factor for renal failure [25]. Some studies have demonstrated a benefit of statins on albuminuria [6, 26, 27], while others failed to indicate such an effect [16, 28]. Our meta-analysis suggested an overall significant decrease of albuminuria after statin therapy (decrease by 0.46, *P* < 0.0001), with the greater improvement of albuminuria among studies with greater baseline albuminuria. Notably, our results are consistent with the meta-analysis performed by Kevin Douglas et al. [29]. The beneficial effect of statins on albuminuria may be potentially explained by cholesterol dependent effects and cholesterol independent effects [30]. Keane et al. [31] have showed that dyslipidemia contributes to glomerular and interstitial injury and the severity of the hypercholesterolemia correlates with albuminuria. In addition, statins may have other cholesterol-independent renoprotective actions, such as reducing neutrophil and macrophage infiltration, up-regulating endothelial nitric oxide (NO) synthase, inhibition of renal cell proliferation, antifibrotic and antioxidant effects, and down-regulation of inflammatory cytokines [32]. Researches suggested that statins reduce albuminuria at least in part by reducing inflammation and fibrosis in the renal interstitium, seemingly through actions on monocyte chemotactic protein-1 and transforming growth factor-β (TGF-β) [33, 34].

A recent review [32] reported that the benefit of statins may depend on the duration of treatment. We also investigated the effect of statin treatment less than 1 year and between 1 and 3 years. A greater decrease of albuminuria was observed in patients received statin therapy for 1 to 3 years compared with those for less than 1 year. It indicated that the beneficial effect of statins on albuminuria may depend on the duration of statin treatment. Additionally, our study found that statins can reduce albuminuria significantly in patients of T2DM with diabetic nephropathy. Diseaes progression, duration of statin therapy and improved renal blood flow are possible relevant factors [35, 36].

Some authors found statins may slow the decline in eGFR [37, 38]. Nikolic et al. [32] suggested an overall significant increase of GFR after statin therapy (increase by 0.29 ml/min/1.73 m^2^, *p* = 0.04), with the greatest GFR improvement after between 1 and 3 years of statin therapy (0.50 ml/min/1.73 m^2^; *p* < 0.0001). However, our meta-analysis found that statins did not improve eGFR significantly. Just as Satirapoj said [30], as a post hoc analysis, using estimates of renal function, some limitations were observed in interpreting these data, so a small proportion of patients, who had DN, were included in this analysis, whereas our findings in the statin group revealed eGFR did not improve, but no significant decline was observed among DN subjects. Therefore, the available data on statin with eGFR in DN patients are still conflicting, because of possible outcome reporting bias.

Findings from our meta-analysis revealed that statins could reduce both albuminuria and the rate of progression of diabetic nephropathy. The benefits appear to supplement those derived from treatment with renin-angiotensin system (RAS) inhibitors [10]. The pathogenesis of diabetic nephropathy is multifactorial and its precise mechanisms of action remain unclear. However, it is now widely accepted that the rate of functional deterioration correlates with the degree of the renal tubulointerstitial fibrosis [36]. Statins is effective in protecting against tubulointerstitial injury [10] and may slow down the progression of diabetic nephropathy, but this needs to be further validated in large-scale and long follow-up period randomized controlled trials.

As with any study, our meta-analysis had some limitations. Frist, the number of randomized controlled trials was small and only published data included. Second, the detection technique of albuminuria was different. Third, the results are heavily based on the findings of the CARDS trial, which represents more than 90 % of the population of the meta-analysis. So, more clinical researches with larger sample, higher quality and strictly RCT study should be taken in the future.

## Conclusion

Statins reduce albuminuria and UAER significantly . The beneficial effect of statins on renal function is time dependent and better in type 2 diabeties patients with nephropathy. Our findings, though exciting, still require larger and high-quality studies to confirm the kidney benefit of statins in patients with diabetes.
